# Plasma p-tau217 in Alzheimer’s disease: Lumipulse and ALZpath SIMOA head-to-head comparison

**DOI:** 10.1093/brain/awae368

**Published:** 2024-12-16

**Authors:** Andrea Pilotto, Virginia Quaresima, Chiara Trasciatti, Chiara Tolassi, Diego Bertoli, Cristina Mordenti, Alice Galli, Andrea Rizzardi, Salvatore Caratozzolo, Andrea Zancanaro, José Contador, Oskar Hansson, Sebastian Palmqvist, Giovanni De Santis, Henrik Zetterberg, Kaj Blennow, Duilio Brugnoni, Marc Suárez-Calvet, Nicholas J Ashton, Alessandro Padovani

**Affiliations:** Neurology Unit, Department of Clinical and Experimental Sciences, University of Brescia, Brescia 25123, Italy; Department of continuity of care and frailty, Neurology Unit, ASST Spedali Civili Hospital, Brescia 25123, Italy; Neurobiorepository and Laboratory of advanced biological markers, University of Brescia and ASST Spedali Civili Hospital, Brescia 25123, Italy; Neurology Unit, Department of Clinical and Experimental Sciences, University of Brescia, Brescia 25123, Italy; Department of continuity of care and frailty, Neurology Unit, ASST Spedali Civili Hospital, Brescia 25123, Italy; Neurobiorepository and Laboratory of advanced biological markers, University of Brescia and ASST Spedali Civili Hospital, Brescia 25123, Italy; Residency Program in Clinical Pathology and Clinical Biochemistry, Department of Molecular and Translational Medicine, University of Brescia, Brescia 25123, Italy; Department of Clinical Laboratory, ASST Spedali Civili Hospital, Brescia 25123, Italy; Neurology Unit, Department of Clinical and Experimental Sciences, University of Brescia, Brescia 25123, Italy; Department of continuity of care and frailty, Neurology Unit, ASST Spedali Civili Hospital, Brescia 25123, Italy; Neurobiorepository and Laboratory of advanced biological markers, University of Brescia and ASST Spedali Civili Hospital, Brescia 25123, Italy; Residency Program in Clinical Pathology and Clinical Biochemistry, Department of Molecular and Translational Medicine, University of Brescia, Brescia 25123, Italy; Neurology Unit, Department of Clinical and Experimental Sciences, University of Brescia, Brescia 25123, Italy; Department of continuity of care and frailty, Neurology Unit, ASST Spedali Civili Hospital, Brescia 25123, Italy; Neurobiorepository and Laboratory of advanced biological markers, University of Brescia and ASST Spedali Civili Hospital, Brescia 25123, Italy; Residency Program in Clinical Pathology and Clinical Biochemistry, Department of Molecular and Translational Medicine, University of Brescia, Brescia 25123, Italy; Department of Clinical Laboratory, ASST Spedali Civili Hospital, Brescia 25123, Italy; Department of Clinical Laboratory, ASST Spedali Civili Hospital, Brescia 25123, Italy; Neurology Unit, Department of Clinical and Experimental Sciences, University of Brescia, Brescia 25123, Italy; Department of continuity of care and frailty, Neurology Unit, ASST Spedali Civili Hospital, Brescia 25123, Italy; Neurology Unit, Department of Clinical and Experimental Sciences, University of Brescia, Brescia 25123, Italy; Department of continuity of care and frailty, Neurology Unit, ASST Spedali Civili Hospital, Brescia 25123, Italy; Neurology Unit, Department of Clinical and Experimental Sciences, University of Brescia, Brescia 25123, Italy; Department of continuity of care and frailty, Neurology Unit, ASST Spedali Civili Hospital, Brescia 25123, Italy; Neurology Unit, Department of Clinical and Experimental Sciences, University of Brescia, Brescia 25123, Italy; Department of continuity of care and frailty, Neurology Unit, ASST Spedali Civili Hospital, Brescia 25123, Italy; Barcelonaβeta Brain Research Center, Pasqual Maragall Foundation, Barcelona 08005, Spain; Department of Neurology, Hospital del Mar Research Institute, Barcelona 08005, Spain; Cognitive Decline Unit, Department of Neurology, Hospital del Mar, Barcelona 08005, Spain; Department of Clinical Sciences, Clinical Memory Research Unit, Malmö 205 02, Sweden; Memory Clinic, Skåne University Hospital, Malmö 205 02, Sweden; Clinical Memory Research Unit, Department of Clinical Sciences Malmö, Faculty of Medicine, Lund University, Malmö 205 02, Sweden; Department of Clinical Sciences, Clinical Memory Research Unit, Malmö 205 02, Sweden; Memory Clinic, Skåne University Hospital, Malmö 205 02, Sweden; Clinical Memory Research Unit, Department of Clinical Sciences Malmö, Faculty of Medicine, Lund University, Malmö 205 02, Sweden; Department of Psychiatry and Neurochemistry, Institute of Neuroscience and Physiology, The Sahlgrenska Academy, University of Gothenburg, Gothenburg 405 30, Sweden; Department of Psychiatry and Neurochemistry, Institute of Neuroscience and Physiology, The Sahlgrenska Academy, University of Gothenburg, Gothenburg 405 30, Sweden; Clinical Neurochemistry Laboratory, Sahlgrenska University Hospital, Mölndal 431 30, Sweden; Dementia Research Center, Institute of Neurology, University College London, London WC1E 6BT, UK; UK Dementia Research Institute, University College London, London WC1E 6BT, UK; Hong Kong Center for Neurodegenerative Diseases, Hong Kong, China; Wisconsin Alzheimer’s Disease Research Center, University of Wisconsin School of Medicine and Public Health, University of Wisconsin-Madison, Madison, WI 53707, USA; Department of Psychiatry and Neurochemistry, Institute of Neuroscience and Physiology, The Sahlgrenska Academy, University of Gothenburg, Gothenburg 405 30, Sweden; Paris Brain Institute, ICM, Pitié-Salpêtrière Hospital, Sorbonne University, Paris 75013, France; Neurodegenerative Disorder Research Center, Division of Life Sciences and Medicine, and Department of Neurology, Institute on Aging and Brain Disorders, University of Science and Technology of China and First Affiliated Hospital of USTC, Hefei 230001, P.R. China; Department of Clinical Laboratory, ASST Spedali Civili Hospital, Brescia 25123, Italy; Barcelonaβeta Brain Research Center, Pasqual Maragall Foundation, Barcelona 08005, Spain; Department of Neurology, Hospital del Mar Research Institute, Barcelona 08005, Spain; Cognitive Decline Unit, Department of Neurology, Hospital del Mar, Barcelona 08005, Spain; Department of Psychiatry and Neurochemistry, Institute of Neuroscience and Physiology, The Sahlgrenska Academy, University of Gothenburg, Gothenburg 405 30, Sweden; Banner Sun Health Research Institute, Sun City, AZ 85351, USA; Banner Alzheimer’s Institute, Phoenix, AZ 85006, USA; Brain Health Center, University of Brescia, Brescia 25123, Italy; Neurology Unit, Department of Clinical and Experimental Sciences, University of Brescia, Brescia 25123, Italy; Department of continuity of care and frailty, Neurology Unit, ASST Spedali Civili Hospital, Brescia 25123, Italy; Neurobiorepository and Laboratory of advanced biological markers, University of Brescia and ASST Spedali Civili Hospital, Brescia 25123, Italy; Brain Health Center, University of Brescia, Brescia 25123, Italy

**Keywords:** plasma markers, Alzheimer’s disease, p-tau217, Lumipulse, ALZPath, SIMOA

## Abstract

Plasma phosphorylated-tau217 (p-tau217) has been shown to be one of the most accurate diagnostic markers for Alzheimer’s disease. No studies have compared the clinical performance of p-tau217 as assessed by the fully automated Lumipulse and single molecule array (SIMOA) AlZpath p-tau217.

The study included 392 participants, 162 with Alzheimer’s disease, 70 with other neurodegenerative diseases with CSF biomarkers and 160 healthy controls. Plasma p-tau217 levels were measured using the Lumipulse and ALZpath SIMOA assays. The ability of p-tau217 assessed by both techniques to discriminate Alzheimer’s disease from other neurodegenerative diseases and controls was investigated using receiver operating characteristic analyses.

The p-tau217 levels measured by the two techniques demonstrated a strong correlation, showing a consistent relationship with CSF p-tau181 levels. In head-to-head comparison, Lumipulse and SIMOA showed similar diagnostic accuracy for differentiating Alzheimer’s disease from other neurodegenerative diseases [area under the curve (AUC) 0.952, 95% confidence interval (CI) 0.927–0.978 versus 0.955, 95% CI 0.928–0.982, respectively] and healthy controls (AUC 0.938, 95% CI 0.910–0.966 and 0.937, 95% CI 0.907–0.967 for both assays).

This study demonstrated the high precision and diagnostic accuracy of p-tau217 for the clinical diagnosis of Alzheimer’s disease using fully automated or semi-automated techniques.

## Teaser

Pilotto *et al.* compared the ability of the Simoa® ALZpath and Lumipulse® plasma p-tau217 assays to discriminate Alzheimer's disease from other neurodegenerative diseases and healthy controls. The results showed strong concordance between the two techniques, with both displaying high diagnostic accuracy.

**See Toniolo (https://doi.org/10.1093/brain/awaf007) for a scientific commentary on this article.**

## Introduction

CSF biomarkers are informative, sensitive and specific for the detection of Alzheimer’s disease (AD) in clinical and research settings from early stages of the disease.^1,2^ The recent development of plasma biomarkers is dramatically changing the AD scenario, as they are scalable tools to aid clinical evaluation and trial recruitment.^3,4^ Phosphorylated tau (p-tau) species stand at the forefront of emerging AD blood tests, exhibiting superior accuracy in diagnosis and specificity for the disease compared to the amyloid-beta (Aβ) 42/40 ratio or other suggested biomarkers.^5-9^

To date, phosphorylated tau at threonine 217 (p-tau217) appeared to be one of the most sensitive and specific AD markers compared to other p-tau species for differentiating AD from other neurodegenerative disorders.^6,10-16^

In addition, p-tau217 exhibits a unique longitudinal trajectory in preclinical AD amyloid-positive individuals, with increases over time being significantly associated with worsening cortical atrophy and declining cognitive performance.^4,6,13,17,18^

Most published studies focusing on p-tau species have used immunoassays on either the Meso Scale Discovery (MSD) or single molecule array (SIMOA) platforms.^5,10,11,15,19^ The recent development of similar assays using chemiluminescent enzyme immunoassay (CLEIA) technology (including the fully automated Lumipulse platform) represents an attractive further step for their easier use and wider consistent applicability in clinical practice. The fully automated platform produces more consistent results between laboratories and overtime in the same laboratory.

For Lumipulse p-tau217, only one preliminary study suggested a high discrimination accuracy for AD diagnosis, though without a head-to-head comparison available to date.^20^ Despite the growing amount of preprint data available, there is an urgent need for high-quality technical and clinical validation of newly developed p-tau217 markers.

The objective of the study was therefore to compare the diagnostic accuracy performance of Lumipulse versus SIMOA plasma p-tau217 in a large real-world memory clinic scenario with clinically approved CSF AD biomarkers as the reference standard.

## Material and methods

### Study population

The study included participants with mild cognitive impairment (MCI) or mild dementia who underwent CSF assessment at the outpatient Neurodegenerative clinic of the Brescia University Hospital, Italy, and age- and sex-matched healthy control (HC) subjects. A standardized full cognitive and behavioural assessment, including Mini-Mental State Examination (MMSE), Neuropsychiatric Inventory (NPI) and Clinical Dementia Rating Scale (CDR), as well as an evaluation of comorbidity using the Cumulative Illness Rating Scale (CIRS), was performed in each participant.

Patients were clinically classified as MCI, dementia with Lewy bodies (DLB),^21^ MCI associated with motor neuron disease^22^ or behavioural frontotemporal dementia (FTD).^23^ The diagnosis of AD was carried out clinically and confirmed biologically according to CSF AD-pattern Aβ_42_/p-tau181 ratio >11.1.^24-26^ Subjects with clinically defined NDD but AD-related pattern were excluded. A group of neurologically and cognitively normal individuals (HC) was recruited from participants’ caregivers, as part of the Life-BIO cohort. The following exclusion criteria were applied: (i) diagnosis of any neurological disorder; (ii) presence of subjective cognitive complaints; (iii) normal neurological examination and Montreal Cognitive Assessment screening; (iv) major psychiatric disorder; or (v) recent inflammatory events. The study was approved by the local ethics committee (NP 1471, DMA, Brescia) and performed in conformity with the Declaration of Helsinki; informed consent was obtained from each study participant or their legally authorized representative.

### CSF collection and analyses

Each patient underwent lumbar puncture in fasting condition according to the standardized protocol of the outpatient neurodegenerative clinic. The CSF specimens were collected in 15-ml polypropylene sterile tubes, gently mixed to avoid gradient effects and sent directly to the hospital laboratory for routine assessments and Lumipulse CSF core AD markers.^24^ The internal cut-off value of Lumipulse was Aβ_42_/p-tau181 ratio >11.1; amyloid positivity was additionally evaluated using the Aβ_42_/Aβ_40_ < 0.069 cut-off.

### Plasma collection and analysis

Blood samples were collected from each participant using 7.5 ml tubes containing K2-EDTA. The tubes were gently inverted 5 to 10 times to mix the blood and then centrifuged at 2500*g* for 10 min at room temperature. Next, 0.5-ml plasma aliquots were pipetted into polypropylene cryotubes and directly stored at ultra-low temperature freezing (ULTF) −80°C for both Lumipulse and SIMOA analyses.

On the day of analysis, the plasma samples were brought to room temperature (21°C–23°C). Following the manufacturer’s instructions, plasma samples were centrifuged at 2000*g* for 5 min. The plasma was then transferred to the instrument cuvettes for testing on Lumipulse using the Lumipulse^®^ G p-tau217—Plasma Immunoreaction Cartridges RUO (for research use only) made up of three different components: the anti-phosphorylated tau (217) monoclonal antibody (mouse)-coated particles, antibodies conjugate and assay buffer solution. The reagents are designed for a fully automated chemiluminescent enzyme immunoassay (CLEIA); the limit of detection is 0.030 pg/ml and the dynamic range is 0.030–10 pg/ml.

The commercial ALZpath p-tau217 assay uses a proprietary monoclonal p-tau217 specific capture antibody, an N-terminal detector antibody and a peptide calibrator.^5^ It has been validated as a fit-for-purpose assay^27^ with a limit of detection of 0.0052–0.0074 pg/ml, a functional lower limit of quantification of 0.06 pg/ml and a dynamic range of 0.007–30 pg/ml. The spike recovery for the endogenous analyte was 80%, and intrarun and interrun precision was 0.5%–13% and 9.2%–15.7%, respectively. Before SIMOA testing, the samples were spun at 14 000*g* for 3 min or equivalent to precipitate debris. SIMOA analyses were performed on HD-X with commercially available p-tau217 ALZpath Simoa^®^ pTau-217 V2 Kits (Quanterix) at the Clinical Neurochemistry Laboratory, Sahlgrenska University Hospital, Mölndal, Sweden.^5^

### Testing precision analyses

The study investigated the within-lab precision of the Lumipulse plasma Immunoreaction Cartridges RUO through repeated inter-day testing schemes 3 × 5 and 5 × 5. For the 3 × 5 testing, three plasma aliquots from a healthy control (negative control) and three plasma aliquots from an AD patient (positive control) were used. Two commercial quality control (QC) samples, namely the high (Level 2, L2) and the low (Level 1, L1) levels provided by the company, were tested five times a day for 5 days. The Lumipulse testing precision has been assessed in 15 and 25 runs based on the CLSI EP15.^28^ The 15 independent negative control and positive control plasma samples were stored at −80°C during the 5 days of the assessments. The L1 and L2 controls of the p-tau217 kit were kept at −20°C as per the manufacturer’s instructions.

Outliers were defined based on single values higher/lower than 3 SD compared to the mean of the group.

### Statistical analyses

Normality distribution was evaluated using the Shapiro-Wilk test and Q-Q plots. To compare clinical and demographic characteristics as well as cognitive assessments and CSF and plasma markers between diagnostic groups (AD, HC, NDD), the Kruskal–Wallis test was conducted. The between-group differences in plasma markers were evaluated in a univariate model adjusted for age, sex and CIRS total score. The comparability between the two analytical platforms was assessed using Passing-Bablok regression, while their imprecision was assessed by calculating the laboratory’s coefficient of variation (CV). The association between plasma and CSF biomarkers was determined using Spearman’s correlation coefficient within a correlation matrix.

The accuracy in discriminating between AD and NDD/HC and between subjects with amyloid positivity (using the Aβ_42_/Aβ_40_ ratio) using plasma biomarkers, in terms of specificity and sensibility, was assessed using a receiver operating characteristic (ROC) approach. Area under the ROC curves (AUCs) were computed using the pROC package in R. The same statistical analyses were performed only considering AD-MCI and NDD-MCI subgroups (i.e. CDR <1). All analyses were conducted using R statistical software (https://www.r-project.org/). Statistical significance was defined at α = 0.05, and all tests were two-tailed.

## Results

### Precision and repeatability of p-tau217 Lumipulse G600II testing

Fifteen different specimens of 500 μl were aliquoted from plasma samples collected from one AD CSF-confirmed patient (positive control, PC) and a healthy control subject (negative control), both tested as independent samples to perform the between-day repeatability and calculate the testing precision. For p-tau217, the clinical laboratory and between-run CVs (%) for positive and negative controls were 2.340 and 1.310 for the positive control and 3.749 and 2.280 for the negative control, respectively (Supplementary Tables 1, 2 and 5). Likewise, the commercial QC samples resulted in within-laboratory and between-run CVs of 5.080 and 5.340 for L1, and 3.387 and 3.490 for L2, respectively (Supplementary Tables 3–5).

### Clinical validation and SIMOA head-to-head comparison

**Figure 1:**
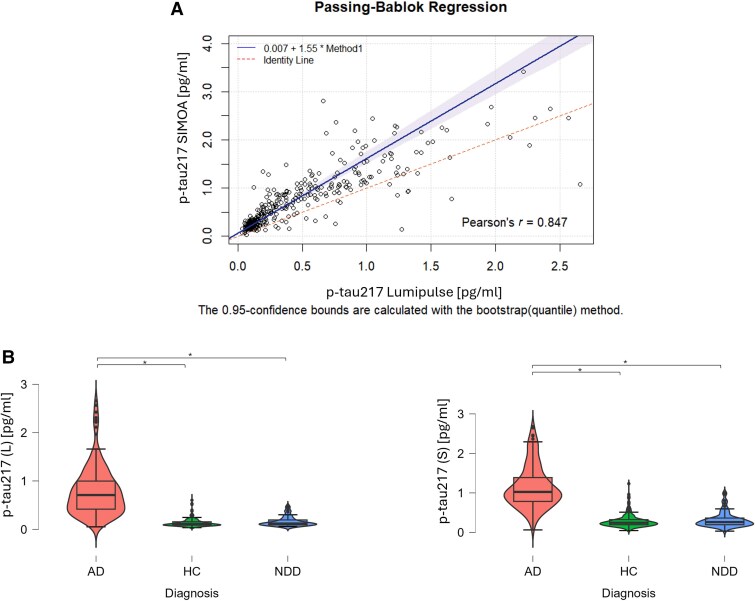
**Plasma p-tau217 levels detected by Lumipulse and SIMOA in the whole cohort and subgroups of participants.** Passing-Bablok regression in **A** shows the comparison between the two testing platforms Lumipulse (L) and SIMOA (S), which highlights a constant, systematic and proportional error between the two detection methods. In **B**, p-tau217 levels in Alzheimer’s disease (AD), healthy control (HC) and non-Alzheimer’s neurodegenerative disorders (NDD) groups measured using Lumipulse (L) and SIMOA (S). p-Tau217 is significantly higher in AD compared with both HC and NDD, for both testing platforms. p-tau217 (L)/(S) = phosphorylated tau 217 tested on Lumipulse (L) and SIMOA (S). SIMOA = single molecule array.

The clinical study included 392 subjects, namely 232 patients and 160 controls. The clinical assessment and CSF AD markers allowed the classification of patients in 162 AD (of which 112 had MCI) and 70 other NDD (of which 45 had MCI) cases (Supplementary Fig. 1). No outliers were detected and all SIMOA and Lumipulse values were included in the final analyses. In the whole cohort and AD/NDD/HC subgroups, no correlations between age or sex and plasma p-tau217 levels (tested by Lumipulse and SIMOA) were detected. Clinical and demographic data and CSF core biomarkers are indicated in Table 1. P-tau217 values showed a constant, systematic and proportional error between the two detection methods as highlighted by the Passing-Bablok regression (Fig. 1). The intercept was 0.067 [95% confidence interval (CI) 0.046–0.084] and the slope = 1.552 (95% CI 1.433–1.703). AD showed higher levels of plasma p-tau217 assessed with both techniques compared to both NDD and HC (Table 1 and Fig. 1).

**Table 1:** Participants’ characteristics and plasma biomarkers assessed by Lumipulse and SIMOA platforms

	HC (*n* = 160)	AD (*n* = 162)	NDD (*n* = 70)	*P*-value	η^2^
Age, years	71.016 (5.450)	72.478 (7.287)	69.593 (7.332)	0.005^a^	0.035
Sex, female:male	101:59	102:60	28:42	<0.001^a^	–
Ethnicity	Caucasian 100%	Caucasian 100%	Caucasian 100%	–	–
MMSE, adjusted score	28.70 (1.0)	24.897 (5.230)	25.667 (4.926)	<0.001^b,c^	0.118
**Comorbidities and medical treatment**					
CIRS, total	1.772 (1.053)	7.746 (6.947)	8.724 (7.430)	<0.001^b,c^	0.323
CIRS, liver	0.081 (0.306)	0.230 (0.459)	0.172 (0.384)	0.009^b,c^	0.031
CIRS, kidney	0.027 (0.211)	0.248 (0.591)	0.276 (0.528)	<0.001^b^	0.056
**AD CSF core biomarkers**					
t-tau, pg/ml	–	696.481 (388.766)	360.904 (257.172)	<0.001	0.159
p-tau181, pg/ml	–	113.943 (61.599)	40.007 (15.040)	<0.001	0.063
Aβ_42_, pg/ml	–	479.332 (165.290)	1060.177 (1051.273)	<0.001	0.412
**Plasma biomarkers**					
Plasma p-tau217 (L), pg/ml	0.181 (0.222)	0.794 (0.511)	0.163 (0.105)	<0.001^a,b^	0.418
Plasma p-tau217 (S), pg/ml	0.353 (0.349)	1.163 (0.565)	0.323 (0.196)	<0.001^a,b^	0.465

Data are expressed as mean and standard deviation. *P*-values show the difference between Alzheimer’s disease (AD) CSF core biomarkers profile groups and were computed with a Mann–Whitney U-test [age, Mini-Mental State Examination (MMSE), AD CSF core biomarkers] or a chi-squared test (sex). Aβ_42_ = amyloid-beta 1–42; CIRS = cumulative index rating scale; HC = healthy control; NDD = non-Alzheimer neurodegenerative disorders; p-tau181 = phosphorylated tau 181 isoform; p-tau217 (L)/(S) = phosphorylated tau 217 tested on Lumipulse (L) and SIMOA (S); t-tau = total tau. SIMOA = single molecule array.

^a^Significant comparison AD versus NDD.

^b^Significant comparison AD versus HC.

^c^Significant comparison NDD versus HC.

The correlation analyses demonstrated a positive relationship between plasma p-tau217 analysed by Lumipulse testing and CSF p-tau181 and t-tau (respectively, ρ = 0.743, *P* < 0.001 and ρ = 0.879, *P* < 0.001). A similar correlation was found for plasma p-tau217 tested by SIMOA and CSF p-tau181 and t-tau (respectively, ρ = 0.688, *P* < 0.001 and ρ = 0.555, *P* < 0.001), being p-tau217 Lumipulse/SIMOA levels highly correlated (ρ = 0.892, *P* < 0.001). p-Tau 217 tested with both Lumipulse and SIMOA negatively correlated with CSF Aβ_42_ levels (ρ = −0.451, *P* < 0.001; ρ = −0.468, *P* < 0.001, respectively).

### Discriminant analyses for Alzheimer’s disease diagnosis

**Figure 2:**
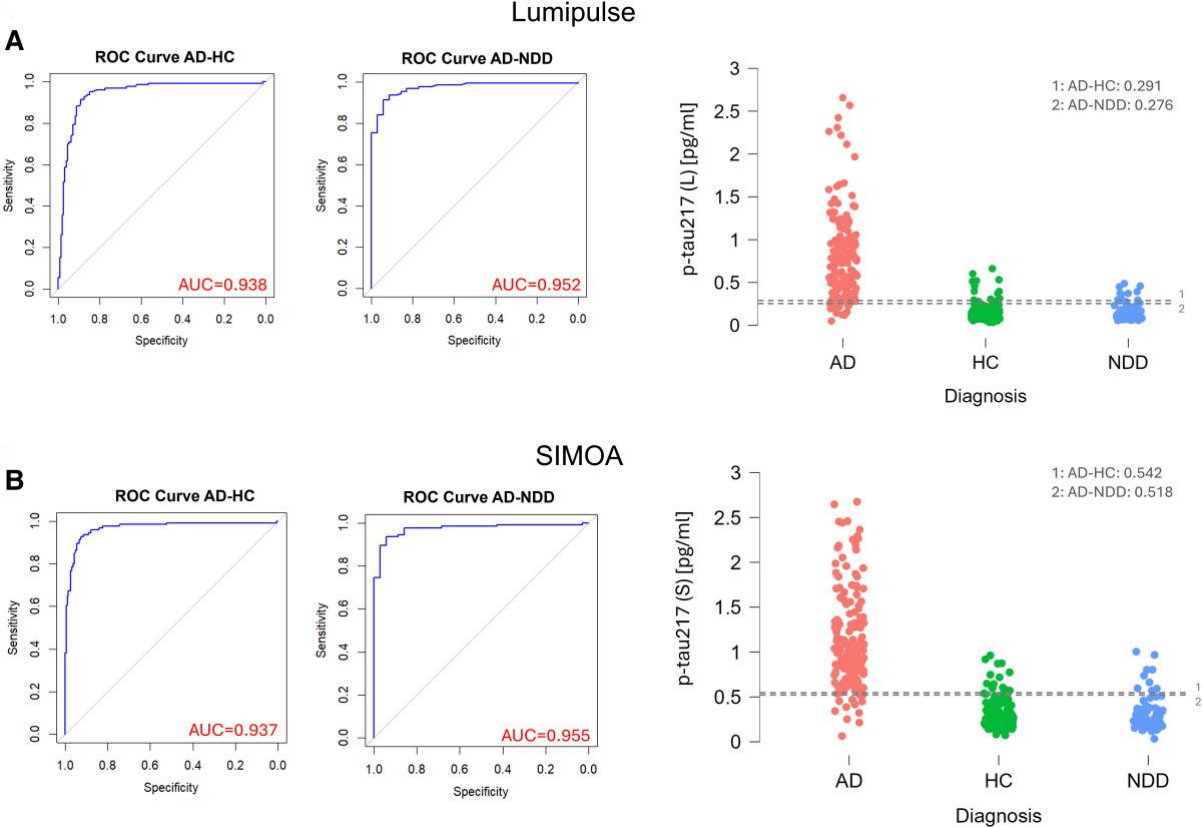
**Diagnostic accuracy of p-tau217 for Alzheimer’s disease diagnosis using Lumipulse and SIMOA.** Alzpath assessment or receiver operating characteristic (ROC) curve of Alzheimer’s disease-healthy control (AD-HC) and Alzheimer’s disease-non-Alzheimer’s neurodegenerative disorders (AD-NDD) populations with the area under the curve (AUC) and optimal Youden cut-off represented by the dashed grey line in the distribution plots for (**A**) Lumipulse (L) and (**B**) SIMOA (S). p-tau217 (L)/(S) = phosphorylated tau 217 tested on Lumipulse (L) and SIMOA (S). SIMOA = single molecule array.

The discriminatory accuracy of plasma biomarkers analysed with Lumipulse and SIMOA techniques for the diagnosis of AD with respect to both HC and NDD was separately evaluated using AUC-ROC analysis (Fig. 2 and Table 2). Plasma p-tau217 analysed on the Lumipulse system resulted in an AUC for AD versus NDD of 0.952 (95% CI 0.927–0.978) and 0.938 (95% CI 0.910–0.966) versus HC.

**Table 2:** Diagnostic accuracy of Lumipulse and SIMOA plasma p-tau217

		AUC	95%CI	Sensitivity	Specificity	Youden cut-off	Fold-change
p-tau217 (L)	AD versus HC	0.938	0.910–0.966	0.882	0.893	0.291	4.387
	AD versus NDD	0.952	0.927–0.978	0.894	0.841	0.276	4.871
p-tau217 (S)	AD versus HC	0.937	0.907–0.967	0.919	0.874	0.542	3.295
	AD versus NDD	0.955	0.928–0.982	0.938	0.887	0.518	3.601

Area under the curve (AUC), 95% confidence intervals (CI), sensitivity, specificity and Youden cut-off for receiver operating characteristic (ROC) analysis on Lumipulse (L) and SIMOA (S) testing. AD = Alzheimer’s disease; HC = healthy control; NDD = non-Alzheimer’s neurodegenerative disorders; p-tau217 (L)/(S) = phosphorylated tau 217 tested on Lumipulse (L) and SIMOA (S). SIMOA = single molecule array.

Plasma p-tau217 tested on SIMOA yielded similar diagnostic accuracy, with an AUC of 0.955 (95% CI 0.928–0.982) for the discrimination of AD from NDD and 0.937 (95% CI 0.907–0.967) from HC. The calculated best cut-offs (i.e highest Youden index) for AD versus HC and AD versus NDD were 0.291 pg/ml and 0.276 pg/ml (Fig. 2), respectively, for Lumipulse. The computed best cut-offs considering p-tau217 levels in SIMOA for AD versus HC and AD versus NDD were 0.542 pg/ml and 0.518 pg/ml, respectively, (highest Youden index).

In the MCI subset, including 112 AD-MCI and 45 NDD-MCI, the AUC and the cut-offs were similar to the whole cohort (Supplementary Tables 6 and 7). Specifically, Lumipulse p-tau217 yielded an AUC of 0.946 (95% CI 0.911–0.981) for discrimination between AD-MCI and NDD-MCI and 0.960 (95% CI 0.936–0.985) for differentiation from HC. SIMOA ALZpath p-tau217 exhibited similar accuracy, with AUCs of 0.934 (95% CI 0.893–0.976) AD-MCI versus NDD-MCI and 0.960 (95% CI 0.936–0.985) for HC (Supplementary Table 7). NDD subjects who resulted positive to p-tau217 Lumipulse (*n* = 11) or SIMOA (*n* = 9) showed similar CSF core AD markers compared to NDD below the established cut-off (Supplementary Table 8). The head-to-head comparison with ALZpath p-tau217 showed a fold-change for Lumipulse equal to 4.387 and 4.871 for AD versus HC and NDD and a fold-change for SIMOA of 3.295 and 3.601, respectively. In the subset of 168 with available CSF Aβ_42_/Aβ_40_ ratio, 116 were amyloid-positive; p-Tau 217 Lumipulse and SIMOA showed AUCs in the ROC analyses of 0.90 and 0.91 for differentiating amyloid positivity, respectively (Supplementary Fig. 2).

## Discussion

This study demonstrated the excellent clinical accuracy of plasma p-tau217 for AD detected using Lumipulse and SIMOA techniques. These findings suggest that both techniques are valid, solid and comparable alternatives for assessing plasma p-tau217 levels, potentially broadening the accessibility of this biomarker in clinical settings.

The technical validation of Lumipulse p-tau217 assessment showed a CV within-laboratory of around 5% for p-tau217 lower concentrations (negative control and L1) and below 3.5% for higher concentrations (positive control and L2). These values are in line with the precision levels observed for both SIMOA and MSD techniques.^5,15^ The method comparison analysis (Passing-Bablok) showed that the two testing platforms identified different but highly related p-tau217 concentrations. Therefore, two distinct cut-offs (or conversion methods) for p-tau217 are required for Lumipulse and SIMOA techniques. This is consistent with previous data evaluating p-tau181 assays across techniques in clinical settings.^24^

When applied in a clinical setting, the p-tau217 plasma assay confirmed its high biological validity, with a high discrimination accuracy of more than 93% for AD compared to other CSF-confirmed patients with NDDs and age-matched HCs. These results are consistent with the greater fold-change of p-tau217 compared to other p-tau species, namely p-tau231 and p-tau181 recently demonstrated.^4,5,10-16^

Of note, the cut-offs resulting in the highest Youden index in the ROC analyses for discriminating AD from NDD and controls resulted in very similar cut-off values across assays, suggesting the possible adoption of a single value for AD diagnosis, ideally to be established by multi-centre validation studies.

The strong correlation between plasma p-tau217 and CSF p-tau181 standard levels further supports its utility as a non-invasive alternative for diagnosing AD, potentially limiting CSF analysis to a subset of subjects with borderline levels.^29^ Of note, the study included subjects with different diseases and ages, without any *a priori* selection, thus confirming the broad applicability of such techniques in real-life settings. Nevertheless, further technical validations of the testing methods are warranted to challenge the stability of biomarkers in different settings, as testing immediately after −80°C storage is not always available. This is particularly important when considering the transition from research to clinical use of such an assay, which is still awaiting the ongoing technical and clinical validation process.^24,30^ While our study demonstrates high concordance between the Lumipulse and SIMOA techniques, further validation efforts are warranted to confirm the biological relevance of plasma p-tau217 as a reliable biomarker for AD in different patient populations and disease stages, as well as a marker of copathology in other clinically-defined diseases even using different AD biological marker combinations (i.e CSF versus imaging methods).

Future research should focus on addressing the remaining validation gaps by using predefined cut-off values and optimizing the clinical utility of plasma p-tau217 assays. Furthermore, longitudinal studies are needed to establish the stability of p-tau217 at the individual level over days/weeks or months. Moreover, further studies are needed to evaluate the prognostic value of plasma p-tau217 in predicting disease progression and treatment response in AD patients, even in combination with other existing plasma biomarkers. In addition, efforts should be made to standardize assay protocols and establish reference ranges for plasma p-tau217 levels to facilitate its integration into routine clinical practice for early detection and monitoring of the AD continuum.

In conclusion, our study adds to the growing body of evidence supporting the utility of plasma p-tau217 as a reliable biomarker for the diagnosis of AD. The validation of Lumipulse p-tau217 highlights its potential to complement existing diagnostic approaches and improve the accuracy of AD detection in clinical practice.

## Supplementary Material

awae368_Supplementary_Data

## Data Availability

The datasets used and analysed during the current study are available from the corresponding author on reasonable request.
