# Supplementary material for: Plasma p-tau217 in Alzheimer’s disease: Lumipulse and ALZpath SIMOA head-to-head comparison
Source: Brain. 2024 Dec 16;148(2):408–15. doi: 10.1093/brain/awae368 (PMC11788209; doi:10.1093/brain/awae368)

Supplementary Table 1. Repeatability test Negative control, NC (3 replicates X 5 testing days)

p-tau217 LUMIPULSE	NC-1	NC-2	NC-3	MEAN	SD	CV(%)
day 1 (pg/mL)	0.170	0.176	0.181	0.176	0.006	3.135
day 2 (pg/mL)	0.189	0.186	0.186	0.187	0.002	0.926
day 3 (pg/mL)	0.189	0.194	0.188	0.190	0.003	1.689
day 4 (pg/mL)	0.184	0.194	0.183	0.187	0.006	3.253
day 5 (pg/mL)	0.177	0.181	0.182	0.180	0.003	1.470

Repeatability test of the Lumipulse G600II using 3 aliquots of a healthy controls (Negative control, NC) tested in 5 different days. p-tau217, phosphorylated tau 217; SD, Standard Deviation; CV, Coefficient of Variation; 95%CI

Supplementary Table 2. Repeatability test Positive control, PC (3 replicates X 5 testing days)

p-tau217 LUMIPULSE	PC-1	PC-2	PC-3	MEAN	SD	CV(%)
day 1 (pg/mL)	0.480	0.494	0.497	0.490	0.009	1.851
day 2 (pg/mL)	0.479	0.479	0.478	0.479	0.001	0.121
day 3 (pg/mL)	0.470	0.479	0.473	0.474	0.005	0.967
day 4 (pg/mL)	0.484	0.497	0.482	0.488	0.008	1.670
day 5 (pg/mL)	0.472	0.462	0.464	0.466	0.005	1.136

Repeatability test of the Lumipulse G600II using 3 aliquots of a patient diagnosed with Alzheimer's Disease (Positive control, PC) tested in 5 different days. p-tau217, phosphorylated tau 217; SD, Standard Deviation; CV, Coefficient of Variation; 95%CI

Supplementary Table 3. Repeatability test Level 1, L1 (5 replicates X 5 testing days)

p-tau217 LUMIPULSE G600 II	L1-1	L1-2	L1-3	L1-4	L1-5	MEAN	SD	CV(%)
day 1 (pg/mL)	0.508	0.457	0.448	0.461	0.465	0.468	0.023	4.989
day 2 (pg/mL)	0.448	0.502	0.504	0.502	0.453	0.482	0.029	5.944
day 3 (pg/mL)	0.439	0.44	0.467	0.498	0.506	0.470	0.031	6.686
day 4 (pg/mL)	0.511	0.503	0.457	0.472	0.495	0.488	0.022	4.608
day 5 (pg/mL)	0.492	0.469	0.469	0.502	0.453	0.477	0.020	4.132

Repeatability test of the Lumipulse G600II using 5 commercial QC available products (Level 1, L1) tested in 5 different days. p-tau217, phosphorylated tau 217; SD, Standard Deviation; CV, Coefficient of Variation; 95%CI

Supplementary Table 4. Repeatability test Level 2, L2 (5 replicates X 5 testing days)

p-tau217 LUMIPULSE G600 II	L2-1	L2-2	L2-3	L2-4	L2-5	MEAN	SD	CV(%)
day 1 (pg/mL)	3.922	3.637	3.668	3.955	3.670	3.770	0.154	4.096
day 2 (pg/mL)	3.675	3.583	3.840	3.738	3.845	3.736	0.112	2.987
day 3 (pg/mL)	3.643	3.9	3.696	3.956	3.984	3.836	0.156	4.065
day 4 (pg/mL)	3.942	3.702	3.864	4.001	3.789	3.860	0.119	3.083
day 5 (pg/mL)	3.815	3.75	3.66	3.959	3.726	3.782	0.113	2.999

Repeatability test of the Lumipulse G600II using 5 commercial QC available products (Level 2, L2) tested in 5 different days. p-tau217, phosphorylated tau 217; SD, Standard Deviation; CV, Coefficient of Variation; 95%CI

Supplementary Table 5. Precision results of the commercial QC level 1 (L1) and level 2 (L2), and positive (PC) and negative (NC) controls

Precision	L1	L2	NC	PC
General mean	0.477	3.797	0.184	0.479
DS Laboratory	0.024	0.129	0.007	0.011
CV within Laboratory	5.080	3.387	3.749	2.340
CV between run	5.340	3.490	2.280	1.310

Supplementary Table 6. Participants' characteristics and plasma biomarkers assessed by Lumipulse (L) and SIMOA (S) platforms for MCI.

	AD (N=112)	NDD (N=45)	p-value
Age	72.698 (6.958)	68.181 (7.456)	0.001
Sex (F:M)	70:42	22:23	0.117
MMSE, adjusted value	25.712(3.571)	28.100 (1.202)	<0.001
AD CSF core biomarkers			
t-tau (pg/mL)	679.783 (319.827)	277.707 (304.062)	<0.001
p-tau181 (pg/mL)	116.227 (61.922)	37.224 (13.079)	<0.001
Aβ42 (pg/mL)	473.747 (154.606)	906.231 (303.042)	<0.001
Plasma biomarkers			
Plasma p-tau217 (L) (pg/mL)	0.751 (0.468)	0.166 (0.103)	<0.001
Plasma p-tau217 (S) (pg/mL)	1.108 (0.554)	0.351 (0.221)	<0.001

Supplementary Table 7. Area Under the Curve (AUC), 95% Confidence Intervals (CI), Sensitivity, Specificity and Youden cut-off for ROC analysis on p-tau217, phosphorylated tau 217 tested on Lumipulse (L) and SIMOA (S) for AD MCI and NDD MCI.

		AUC	95%CI	Sensitivity	Specificity	Youden cut-off
p-tau217 (L)	AD MCI-HC	0.960	0.936-0.985	0.918	0.933	0.251
	AD-NDD MCI	0.946	0.911-0.981	0.882	0.848	0.287
p-tau217 (S)	AD MCI-HC	0.960	0.936-0.985	0.918	0.922	0.524
	AD-NDD MCI	0.934	0.893-0.976	0.864	0.848	0.599

Supplementary Table 8. CSF core biomarkers for Lumipulse and SIMOA between p-tau217 positive vs. negative NDD patients. p-tau217 positivity was established using the best optimal Youden cut-off.

	**NDD p-tau217 neg**	**NDD p-tau217 pos**	**p-value**
**Lumipulse**			
	N=59	N=11	
t-tau (pg/mL)	369.259 (272.773)	316..885 (151.951)	0.539
p-tau181 (pg/mL)	39.433 (13.396)	43.036 (22.435)	0.470
Aβ42 (pg/mL)	1117.841 (1132.884)	756.127 (280.519)	0.299
**SIMOA**			
	N=61	N=9	
t-tau (pg/mL)	368.835 (269.543)	308.033 (150.781)	0.512
p-tau181 (pg/mL)	39.310 (13.569)	44.656 (23.172)	0.324
Aβ42 (pg/mL)	1090.430 (1117.144)	858.489 (370.512)	0.541

Supplementary Figure 1 - Flowchart of patient enrollment and final study sample

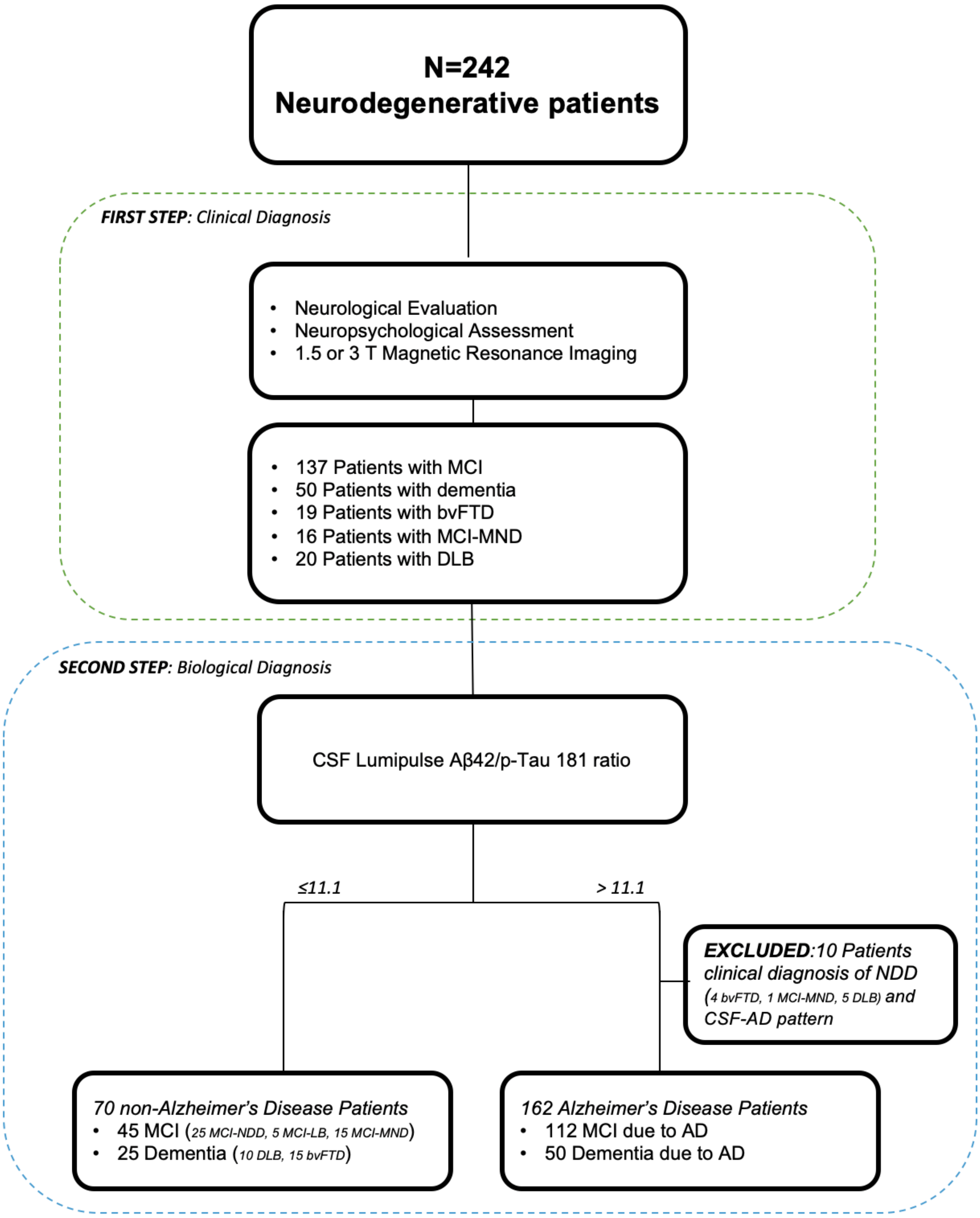

Abbreviations: AD, Alzheimer's Disease; bvFTD, behavioral variant frontotemporal dementia; CSF, Cerebrospinal fluid; DLB, dementia with Lewy bodies; MCI, Mild cognitive impairment; MCI-LB, mild cognitive impairment associated with Lewy bodies disease; MCI-MND, mild cognitive impairment associated with motoneuron disease; MCI-NDD, mild cognitive impairment not associated with biological evidence for Alzheimer's disease pathology; NDD, Neurodegenerative Disease.

Supplementary Figure 2. p-tau217 Lumipulse (L) and SIMOA (S) ROC curves between amyloid positive vs. negative patients. Amyloid positivity was established according to the internal cut-off (Aβ42/Aβ40<0.069).

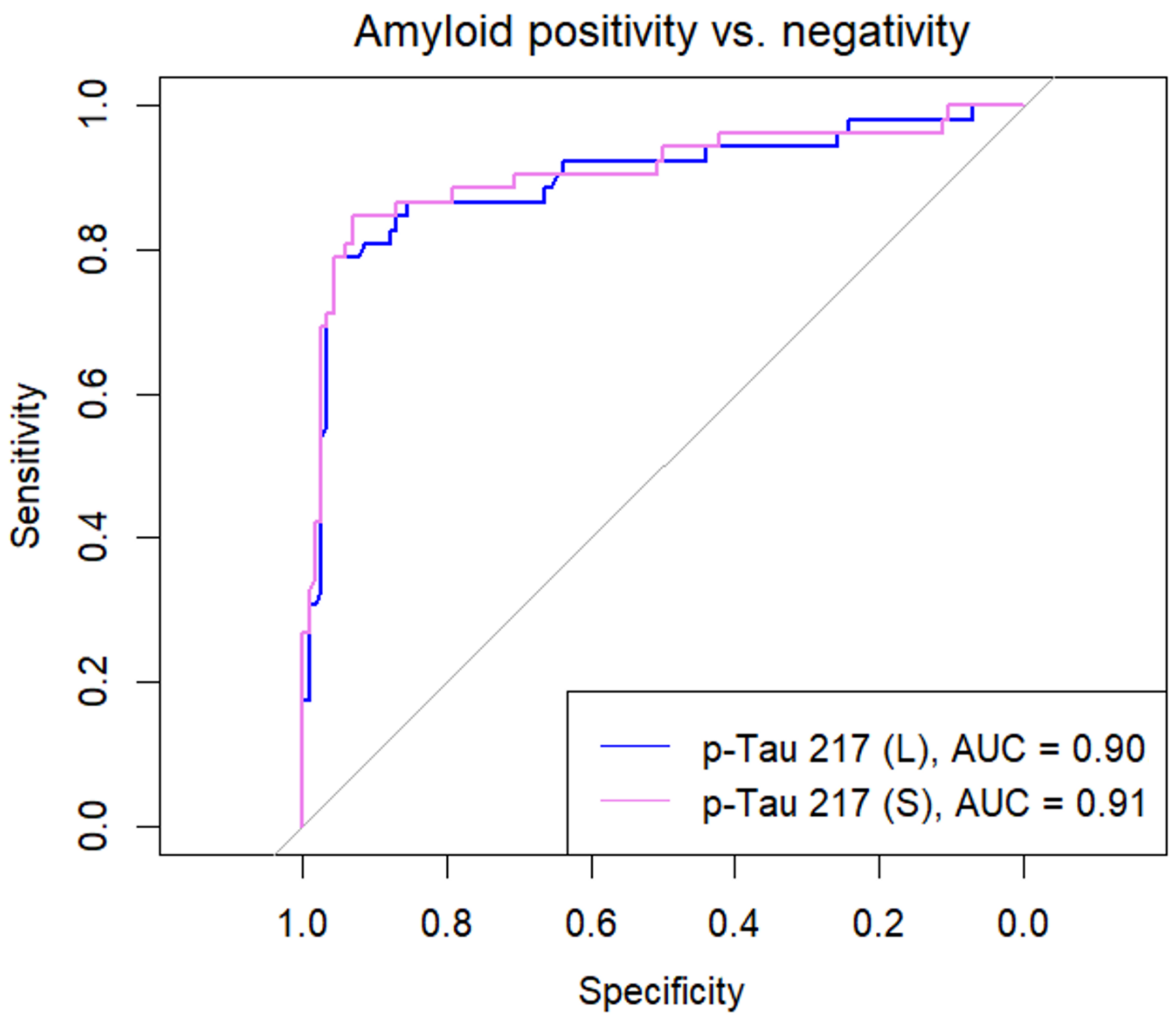

Supplement: awae368_Supplementary_Data [file awae368_supplementary_data.pdf]
